# Serine-129 phosphorylated *α*-synuclein drives mitochondrial dysfunction and calcium dysregulation in Parkinson’s disease model

**DOI:** 10.3389/fnagi.2025.1538166

**Published:** 2025-03-31

**Authors:** Jie Jiao, Weijin Liu, Ge Gao, Hui Yang

**Affiliations:** ^1^Department of Neurobiology, Beijing Key Laboratory of Neural Regeneration and Repair, Beijing Key Laboratory on Parkinson’s Disease, Key Laboratory for Neurodegenerative Disease of the Ministry of Education, School of Basic Medical Sciences, Beijing Institute of Brain Disorders, Collaborative Innovation Center for Brain Disorders, Capital Medical University, Beijing, China; ^2^China Rehabilitation Science Institute, China Rehabilitation Research Center, Beijing Key Laboratory of Neural Injury and Rehabilitation, and School of Rehabilitation Medicine, Capital Medical University, Beijing, China

**Keywords:** *α*-synuclein, phosphorylation, mitochondrial dysfunction, calcium transport, Parkinson’s disease

## Abstract

Phosphorylation of *α*-synuclein at serine-129 (p-*α*-syn) is a hallmark of Parkinson’s disease (PD) and constitutes nearly 90% of α-synuclein in Lewy bodies, playing a critical role in disease progression. Despite its pathological significance, the molecular targets and mechanisms driving p-*α*-syn-induced toxicity, particularly mitochondrial dysfunction, remain poorly understood. In this study, we observed mitochondrial dysfunction in primary cortical neurons derived from mice overexpressing human *α*-synuclein (h-*α*-syn), which also exhibit elevated levels of p-*α*-syn. Notably, inhibiting Ser129 phosphorylation improved mitochondrial function, underscoring the role of p-*α*-syn in mitochondrial damage. To investigate the molecular mechanism, we performed co-immunoprecipitation (CO-IP) combined with mass spectrometry (MS) to identify p-*α*-syn binding proteins. This analysis identified protein tyrosine phosphatase interacting protein 51 (PTPIP51) and vesicle-associated membrane protein-associated protein B (VAPB) as key binding partners. Both proteins are localized in the mitochondria-associated endoplasmic reticulum mem-brane (MAM) and essential for calcium transfer between the endoplasmic reticulum (ER) and mitochondria. Our results showed that p-*α*-syn binds to PTPIP51 and VAPB, disrupting calcium signaling between the ER and mitochondria. Importantly, inhibition of Ser129 phosphorylation partially rescued calcium homeostasis. These findings uncover a novel mechanism by which p-*α*-syn drives mitochondrial dysfunction and calcium dysregulation through its interactions with MAM-associated proteins, providing new insights into its role in PD pathogenesis and potential therapeutic targets.

## 1 Introduction

Parkinson’s disease (PD) is a progressive neurodegenerative disorder primarily affecting individuals over 65 years old, characterized by motor dysfunction and non-motor symptoms (Jankovic and Tan, 2020; Bloem et al., 2021). A hallmark of PD is the accumulation and aggregation of *α*-synuclein (α-syn), a protein that forms toxic oligomers, protofibrils, and ultimately Lewy bodies (LBs) (Beura et al., 2022; Morris et al., 2024; Ye et al., 2023). Under pathological conditions, *α*-syn undergoes hyperphosphorylation at serine 129 (Ser129), with approximately 90% of *α*-syn in PD brains being phosphorylated, compared to less than 4% in healthy controls (Fares et al., 2021; McFarland et al., 2008).

Phosphorylated *α*-synuclein (p-α-syn) not only accelerates α-syn aggregation but also amplifies its neurotoxic effects (Arawaka et al., 2017; Machiya et al., 2010). Elevated p-*α*-syn levels have been detected in cerebrospinal fluid and brain tissues of PD patients, correlating with disease severity (Stewart et al., 2015; Majbour et al., 2016). These findings suggest that p-*α*-syn represents a pathogenic form of *α*-syn and a potential biomarker for disease progression (Kapsali et al., 2024). Mechanistically, p-α-syn disrupts cellular homeostasis by impairing proteasomal and autophagic degradation pathways and altering synaptic transmission (Takaichi et al., 2020). Notably, its co-localization with mitochondria in PD brains and animal models links p-*α*-syn to mitochondrial dysfunction, implicating its role in disrupting mitochondrial processes (Wang et al., 2019).

Despite these insights, the specific molecular targets and mechanisms through which p-*α*-syn contributes to mitochondrial dysfunction remain unclear. Emerging evidence suggests that p-α-syn interacts with mitochondrial proteins or pathways distinct from those affected by non-phosphorylated *α*-synuclein (Parra-Rivas et al., 2023), raising the question of whether it drives pathology through unique mechanisms. Addressing these gaps is crucial for understanding the pathological role of p-α-syn in PD.

In this study, we investigated mitochondrial dysfunction in primary neurons derived from mice overexpressing human *α*-syn (h-α-syn). We observed that p-*α*-syn exacerbates α-syn-induced mitochondrial impairment, while inhibiting Ser129 phosphorylation partially rescued mitochondrial function. To uncover the underlying molecular mechanism, we identified protein tyrosine phosphatase interacting protein 51 (PTPIP51) and vesicle-associated membrane protein-associated protein B (VAPB) as novel interacting partners of p-*α*-syn. Both proteins play key roles in calcium transfer between the ER and mitochondria. Our findings demonstrate that p-*α*-syn disrupts ER-mitochondria calcium homeostasis, providing new insights into its role in mitochondrial dysfunction and neurodegeneration in PD.

## 2 Results

### 2.1 Elevated levels of p-*α*-syn and h-α-syn impair mitochondrial function

**Figure 1:**
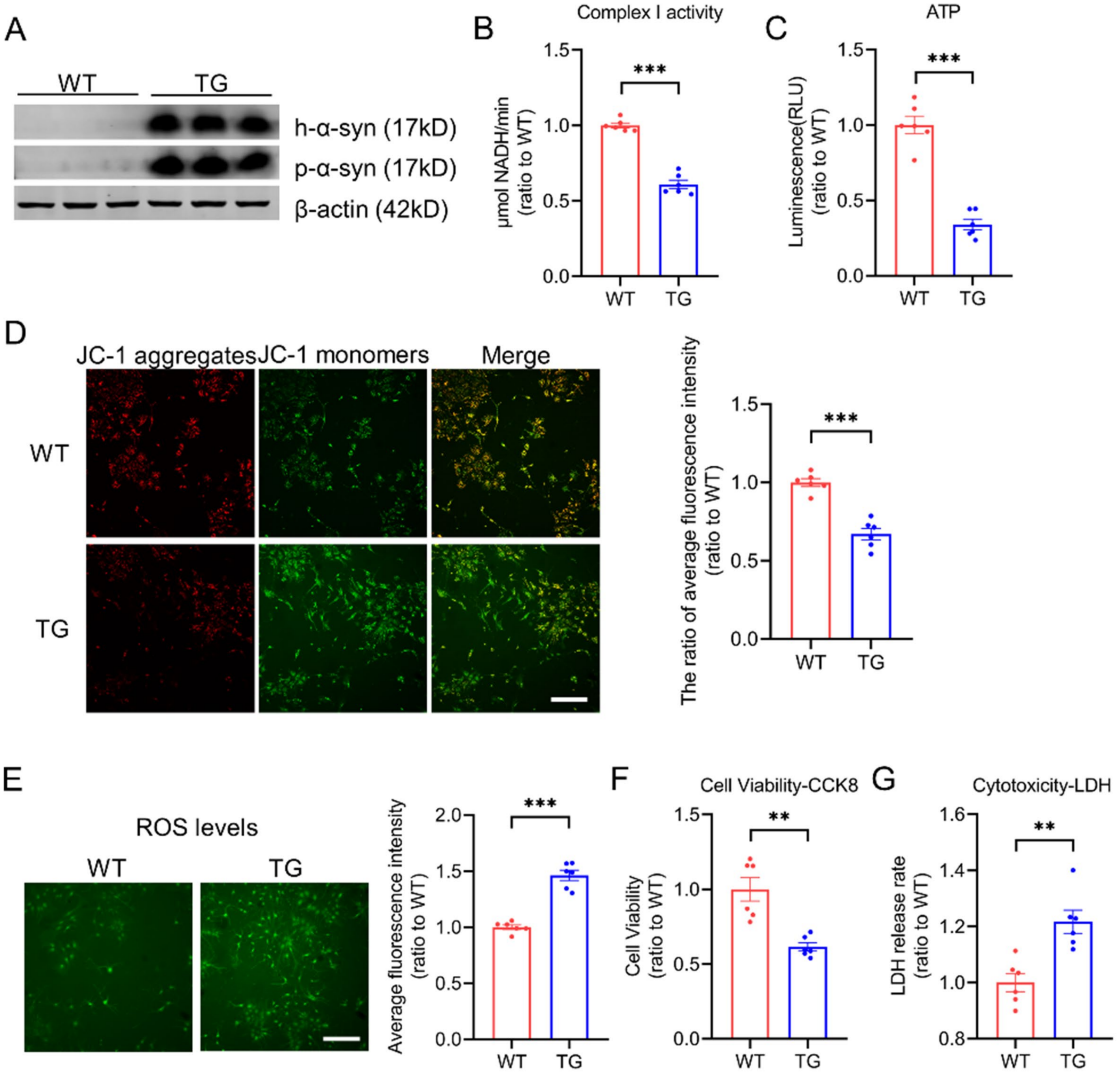
Elevated levels of p-α-syn and h-α-syn impair mitochondrial function. **(A)** Western blot analysis of h-α-syn and p-α-syn expression in primary cortical neurons from WT and TG mice. β-actin was used as a loading control. **(B,C)** Mitochondrial function was assessed by measuring complex I activity **(B)** and ATP levels **(C)** in primary neurons from WT and TG mice (*n* = 6). **(D)** Mitochondrial membrane potential (MMP) was evaluated using JC-1 staining. The ratio of JC-1 aggregates to JC-1 monomers, representing average fluorescence intensity (total fluorescence intensity per cell), is shown on the right (scale bar: 100 μm; *n* = 6). **(E)** Reactive oxygen species (ROS) levels were quantified using the ROS probe H2DCFDA. The fluorescence intensity was normalized to the number of cells and shown on the right (scale bar: 100 μm; *n* = 6). **(F,G)** Cell viability was evaluated using the CCK-8 assay **(F)**, while cytotoxicity was determined by the lactate dehydrogenase (LDH) release assay **(G)** in primary neurons from WT and TG mice (*n* = 6). Data are presented as mean ± standard error of the mean (SEM). Statistical significance was determined using an unpaired t-test (**p < 0.01, *p < 0.001, compared to WT).

To investigate the impact of p-α-syn and h-α-syn on mitochondrial function, we cultured primary cortical neurons from wild-type (WT) mice and transgenic (TG) mice, which overexpress h-*α*-syn and recapitulate both motor and non-motor symptoms of PD (Li et al., 2022). Western blot analysis confirmed overexpression of h-α-syn in TG neurons, accompanied by elevated levels of p-*α*-syn (Figure 1A). Given the link between mitochondrial toxicity and mitochondrial complex I activity (Risiglione et al., 2020), we assessed its functionality in primary neurons. Our results showed that increased p-α-syn and h-*α*-syn levels significantly reduced mitochondrial complex I activity (Figure 1B) and ATP levels (Figure 1C). Additionally, the mitochondrial membrane potential was also reduced (Figure 1D). Furthermore, elevated p-*α*-syn and h-α-syn levels triggered an increase in reactive oxygen species (ROS) generation (Figure 1E), byproducts of mitochondrial respiration that can exacerbate cellular damage. These impairments were associated with decreased cell viability and increased cytotoxicity (Figures 1F,G). Collectively, these findings suggest that p-*α*-syn and h-α-syn disrupt mitochondrial function, resulting in compromised mitochondrial integrity and broader cellular dysfunction.

### 2.2 Inhibition of α-syn phosphorylation at Ser129 improves mitochondrial function

**Figure 2:**
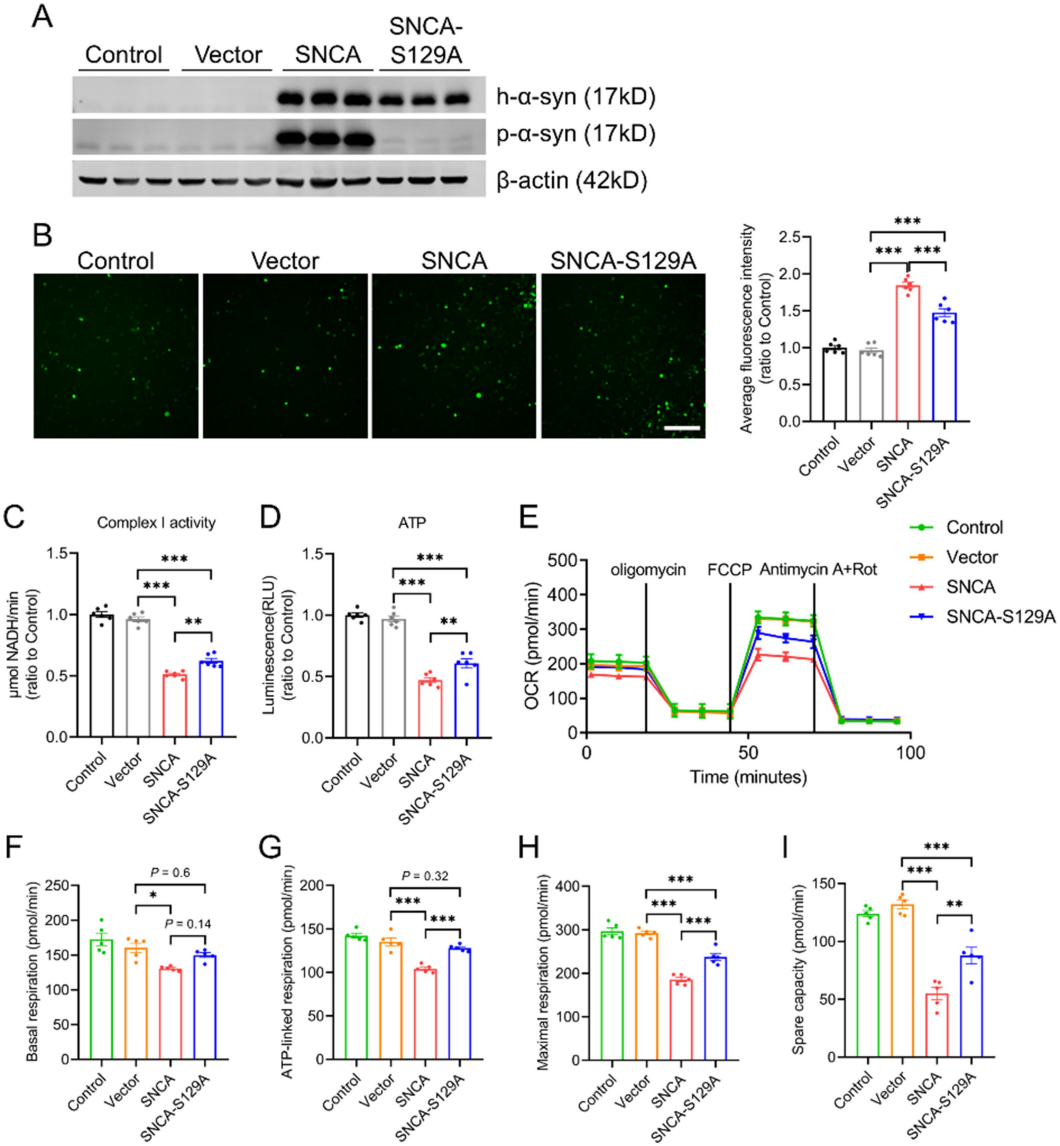
Inhibition of α-syn phosphorylation at Ser129 improves mitochondrial function. SH-SY5Y cells transfected with Vector, SNCA, or SNCA-S129A plasmids for 48 h. **(A)** Western blot analysis of h-α-syn and p-α-syn expression in lysates of SH-SY5Y cells. β-actin was used as a loading control. **(B)** Levels of ROS were assessed using the ROS probe H2DCFDA. Quantitative analysis of average fluorescence intensity (total fluorescence intensity normalized to the number of cells) is displayed on the right (scale bar: 100 μm; *n* = 6). **(C,D)** Mitochondrial complex I activity **(C)** and ATP levels **(D)** were measured in SH-SY5Y cells (*n* = 6). **(E)** Oxygen consumption rate (OCR) was measured in SH-SY5Y cells (*n* = 5). **(F–I)** Mitochondrial respiration parameters derived from OCR data in SH-SY5Y cells, including basal respiration **(F)**, ATP-linked respiration **(G)**, maximal respiration **(H)**, and spare respiratory capacity **(I)** (*n* = 5). Data are expressed as mean ± standard error of the mean (SEM). Statistical analysis was performed using one-way ANOVA (*p < 0.05, **p < 0.01, ***p < 0.001).

As p-α-syn is abundant under pathological conditions and considered a pathogenic form of α-syn, it may play a critical role in mitochondrial damage (Wang et al., 2019). To explore its contribution to mitochondrial dysfunction, we generated a phosphorylation-deficient mutant (SNCA-S129A) by introducing a point mutation at serine 129 in the human SNCA gene. SH-SY5Y cells were transfected with Vector, SNCA, or SNCA-S129A plasmids. Western blot analysis revealed elevated p-α-syn levels in the SNCA group, while phosphorylation was effectively inhibited in the SNCA-S129A group (Figure 2A). Inhibiting Ser129 phosphorylation significantly reduced cellular ROS levels (Figure 2B). Additionally, mitochondrial function was improved in the SNCA-S129A group, as shown by increased mitochondrial complex I activity (Figure 2C) and ATP production (Figure 2D). Furthermore, mitochondrial respiration was assessed using the Seahorse XF system, which showed an increase in the oxygen consumption rate (OCR) when phosphorylation was inhibited (Figure 2E). Specifically, basal respiration, ATP-linked respiration, maximal respiration, and spare respiratory capacity were improved in the SNCA-S129A group (Figures 2F–I). These findings indicate that p-*α*-syn contributes to mitochondrial dysfunction, while inhibiting its phosphorylation can partially restore mitochondrial function.

### 2.3 Identifying proteins interacting with p-α-syn by CO-IP/MS

**Figure 3:**
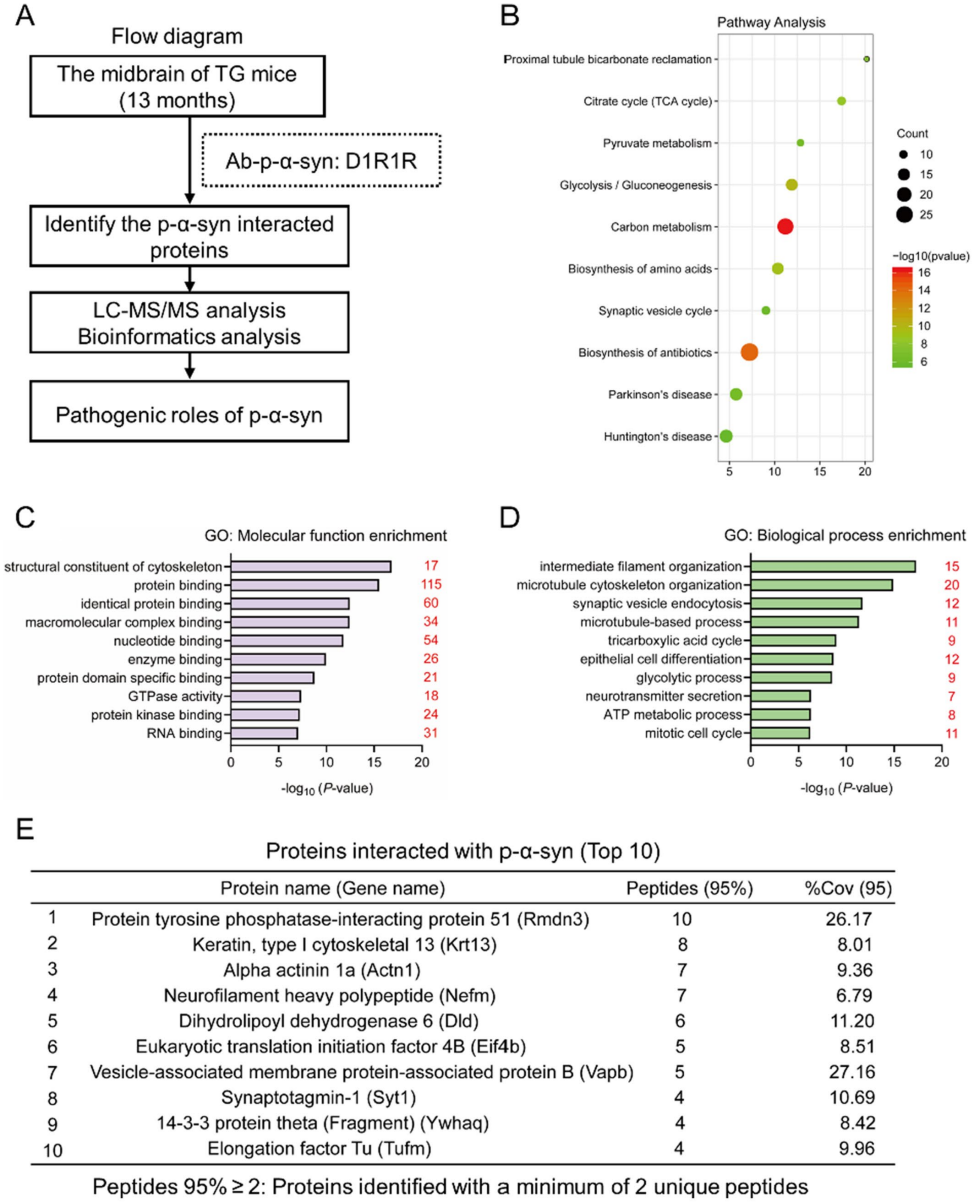
Identifying proteins interacting with p-α-syn by CO-IP/MS. **(A)** Flow diagram of CO-IP with a D1R1R antibody followed by liquid chromatography–tandem mass spectrometry (LC–MS/MS) for the identification of p-α-syn interacting proteins. The analysis was performed using midbrain tissues from 13-month-old TG mice. *n* = 3. **(B)** Kyoto Encyclopedia of Genes and Genomes (KEGG) pathway analysis of p-α-syn interacting proteins. Pathways are represented by circles, with their size corresponding to the number of enriched genes. Low q-values are shown in green, and high q-values are in red. **(C,D)** Gene Ontology (GO) analysis of p-α-syn interacting proteins. GO-Molecular Function (GO-MF) **(C)** and GO-Biological Process (GO-BP) **(D)** terms are displayed, with red numbers indicating the number of interacting proteins associated with each term. **(E)** Top 10 proteins interacting with p-α-syn identified by CO-IP/MS, with PTPIP51 ranked first and VAPB ranked seventh.

To explore the molecular mechanisms underlying p-α-syn-mediated mitochondrial regulation, we performed co-immunoprecipitation (CO-IP) followed by mass spectrometry (MS) to identify p-α-syn-interacting proteins. Using midbrain tissues from 13-month-old TG mice and a p-*α*-syn-specific antibody (D1R1R), we conducted CO-IP experiments and selected proteins identified by at least two peptides with ≥95% confidence for further analysis (Figure 3A). The identified proteins were characterized through KEGG pathway and gene ontology (GO) enrichment analyses using the DAVID Bioinformatics Resources platform. KEGG analysis revealed enrichment in pathways related to carbon metabolism, glycolysis/gluconeogenesis, the citrate cycle (TCA cycle), and PD pathways (Figure 3B). GO molecular function (MF) analysis highlighted structural constituents of the cytoskeleton, protein binding, and identical protein binding as the top categories (Figure 3C), while biological process (BP) analysis emphasized intermediate filament organization, microtubule-based processes, and synaptic vesicle endocytosis (Figure 3D). These analyses provide a comprehensive view of p-*α*-syn’s potential roles in cellular and mitochondrial processes.

Among the identified proteins, PTPIP51 ranked first (Figure 3E). PTPIP51 is a mitochondria-localized protein, particularly enriched at mitochondria-associated membranes (MAMs), where it forms a complex with VAPB to facilitate calcium transfer from the endoplasmic reticulum (ER) to mitochondria (De Vos et al., 2012; Gómez-Suaga et al., 2019). This calcium transfer is critical for ATP production and mitochondrial metabolism (Jiang et al., 2024). Notably, VAPB, ranked seventh among the identified proteins (Figure 3E), was also suggested to interact with p-*α*-syn. These MS findings suggest that p-*α*-syn may contribute to mitochondrial dysfunction through its interaction with PTPIP51 and VAPB, providing potential molecular targets for further investigation.

### 2.4 P-*α*-syn interacts with PTPIP51

**Figure 4:**
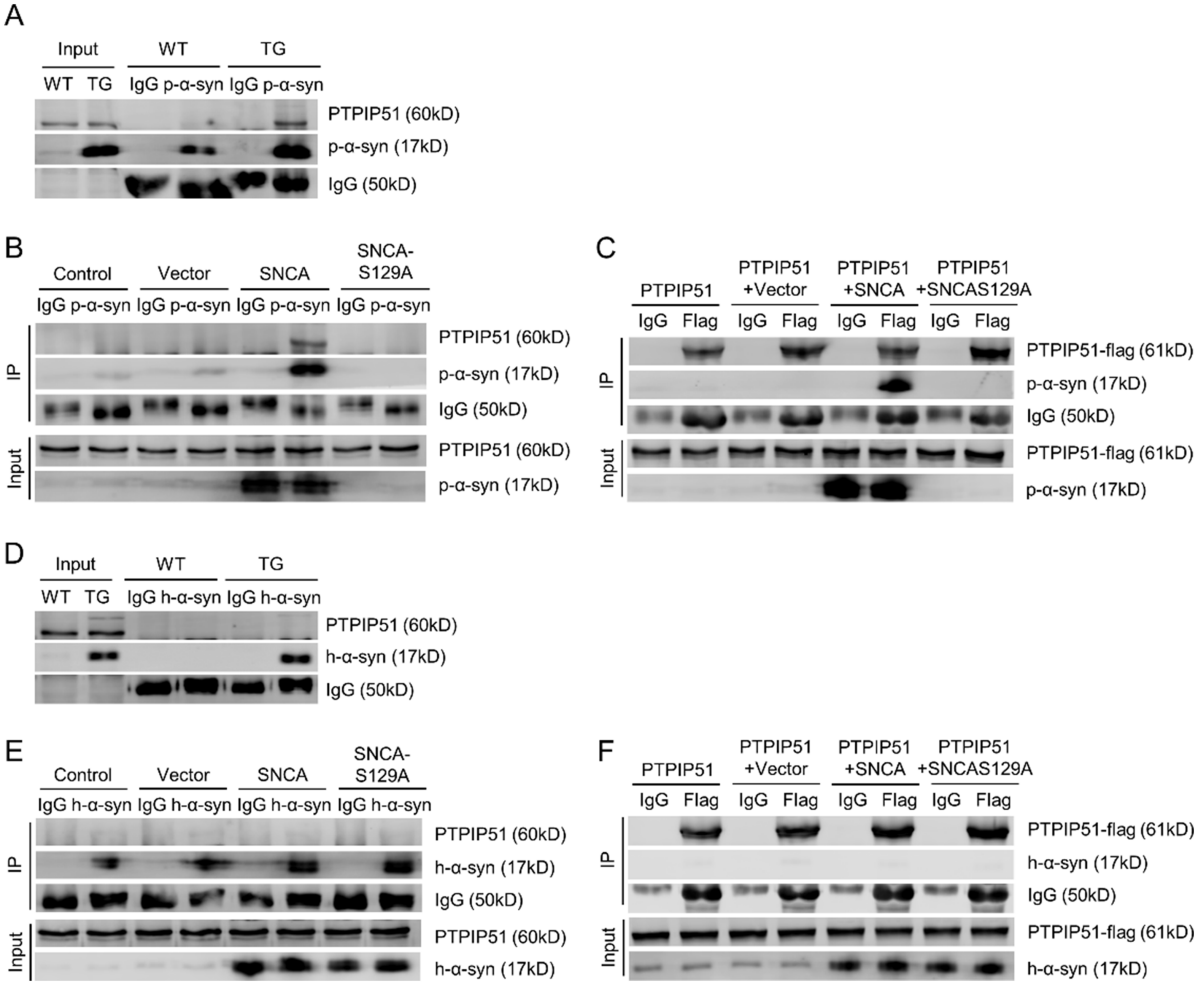
P-α-syn interacts with PTPIP51. **(A)** CO-IP analysis showing the interaction between p-α-syn and PTPIP51 in brain tissues from 13-month-old WT and TG mice using a p-α-syn antibody. **(B)** CO-IP analysis showing the interaction between p-α-syn and PTPIP51 in SH-SY5Y cells transfected with Vector, SNCA, or SNCA-S129A plasmids for 48 h, using a p-α-syn antibody. **(C)** Interaction between p-α-syn and PTPIP51 in SH-SY5Y cells co-transfected with PTPIP51-Flag and Vector, SNCA, or SNCA-S129A plasmids for 48 h, assessed by CO-IP with a Flag antibody. **(D)** CO-IP analysis of the interaction between h-α-syn and PTPIP51 in brain tissues of 13-month-old WT and TG mice using an h-α-syn antibody. **(E)** Interaction between h-α-syn and PTPIP51 in SH-SY5Y cells transfected with Vector, SNCA, or SNCA-S129A plasmids for 48 h, evaluated by CO-IP with an h-α-syn antibody. **(F)** CO-IP experiments showing the interaction between h-α-syn and PTPIP51 in SH-SY5Y cells co-transfected with PTPIP51-Flag and Vector, SNCA, or SNCA-S129A plasmids for 48 h, using a Flag antibody. In all CO-IP experiments, homologous IgG was used as the experimental control.

To determine the interaction between p-*α*-syn and PTPIP51, we performed CO-IP analysis. Using midbrain tissues from 13-month-old WT and TG mice and a p-*α*-syn-specific antibody, we observed an interaction between p-α-syn and PTPIP51 (Figure 4A). This interaction was further examined in cellular models by transfecting SH-SY5Y cells with Vector, SNCA, or SNCA-S129A plasmids. CO-IP results showed the interaction between p-*α*-syn and PTPIP51 in the SNCA group, which was abolished when phosphorylation at serine 129 was inhibited in the SNCA-S129A group (Figure 4B). To validate these findings, we transfected cells with PTPIP51-flag plasmid and conducted CO-IP experiments using an anti-Flag antibody. The results confirmed that PTPIP51 interacts with p-*α*-syn, and this interaction was eliminated upon inhibition of phosphorylation (Figure 4C).

To assess whether this interaction is unique to phosphorylation-modified *α*-syn, we investigated the binding between unmodified h-α-syn and PTPIP51. CO-IP analysis showed that h-α-syn did not interact with PTPIP51 in either TG mouse brain tissue or cellular models (Figures 4D,E). Additionally, even with overexpression of PTPIP51-flag, no interaction with h-*α*-syn was observed (Figure 4F). These results demonstrate that p-α-syn specifically interacts with PTPIP51 in a phosphorylation-dependent manner, highlighting the critical role of Ser129 phosphorylation in mediating this interaction.

### 2.5 P-*α*-syn interacts with VAPB, as does unphosphorylated h-α-syn

**Figure 5:**
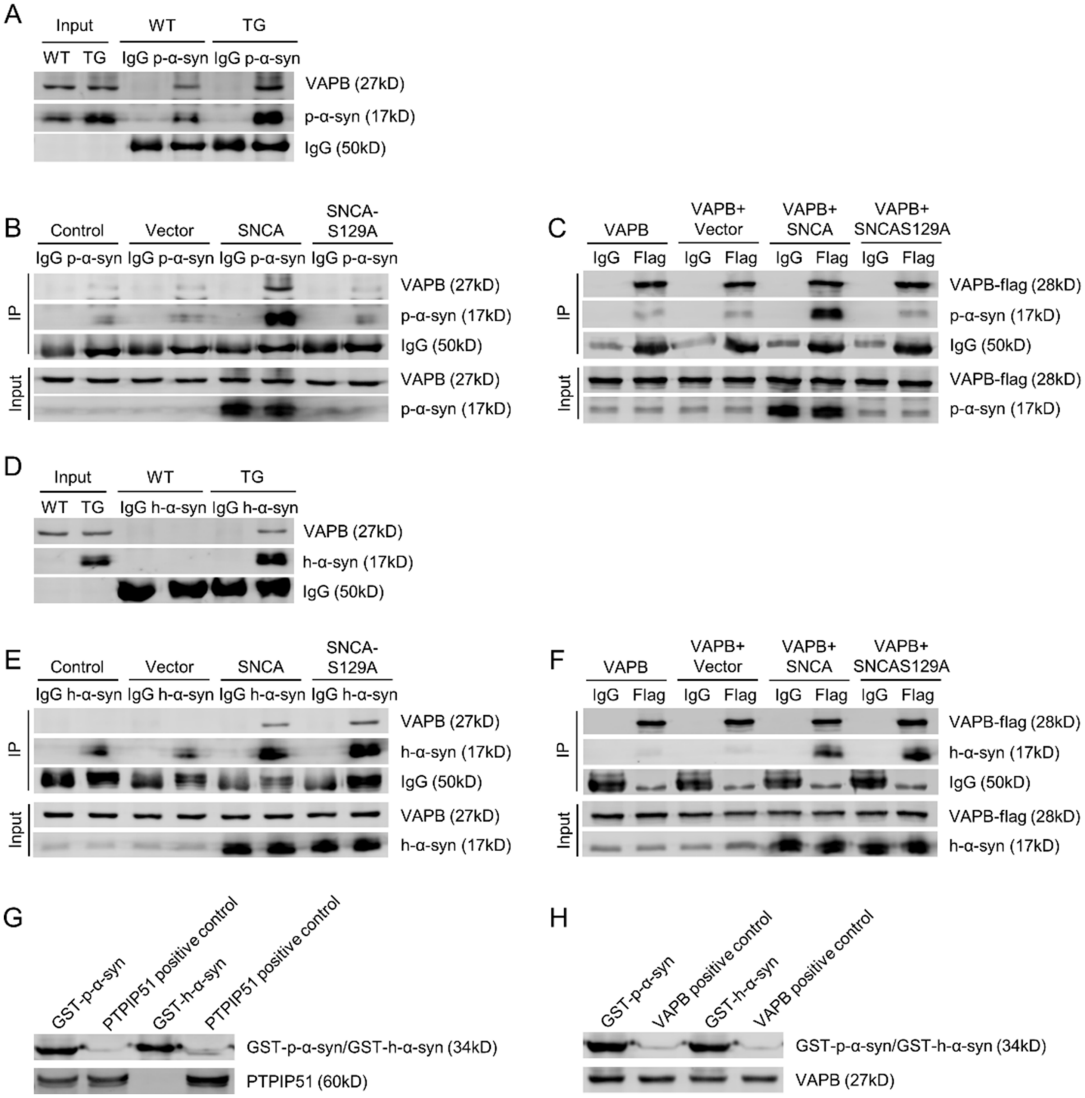
P-α-syn interacts with VAPB, as does unphosphorylated h-α-syn. **(A)** Interaction between p-α-syn and VAPB in brain tissues of 13-month-old WT and TG mice, analyzed by CO-IP using a p-α-syn antibody. **(B)** Interaction between p-α-syn and VAPB in SH-SY5Y cells transfected with Vector, SNCA, or SNCA-S129A plasmids for 48 h, analyzed by CO-IP using a p-α-syn antibody. **(C)** Interaction between p-α-syn and VAPB in SH-SY5Y cells co-transfected with VAPB-Flag and Vector, SNCA, or SNCA-S129A plasmids for 48 h, analyzed by CO-IP using a Flag antibody. **(D)** CO-IP analysis of the interaction between h-α-syn and VAPB in brain tissues of 13-month-old WT and TG mice using an h-α-syn antibody. **(E)** Interaction between h-α-syn and VAPB in SH-SY5Y cells transfected with Vector, SNCA, or SNCA-S129A plasmids for 48 h, assessed by CO-IP using an h-α-syn antibody. **(F)** CO-IP analysis demonstrating the interaction between h-α-syn and VAPB in SH-SY5Y cells co-transfected with VAPB-Flag and Vector, SNCA, or SNCA-S129A plasmids for 48 h, using a Flag antibody. In all CO-IP experiments, homologous IgG was used as the experimental control. **(G,H)** Interactions between GST-h-α-syn, GST-p-α-syn, PTPIP51 **(G)**, and VAPB (H) proteins were investigated using GST-pulldown assays. PTPIP51 and VAPB proteins served as positive controls.

Following the confirmation of p-*α*-syn interaction with PTPIP51, we next examined its interaction with VAPB. Using midbrain tissues from 13-month-old WT and TG mice with a p-*α*-syn-specific antibody, CO-IP analysis revealed an interaction between p-*α*-syn and VAPB (Figure 5A). To further validate this interaction, SH-SY5Y cells were transfected with Vector, SNCA, or SNCA-S129A. CO-IP analysis showed that p-*α*-syn interacts with VAPB in the SNCA group, but this interaction was abolished when phosphorylation at Ser129 was inhibited in the SNCA-S129A group (Figure 5B). Similarly, CO-IP using an anti-Flag antibody in cells transfected with VAPB-flag confirmed the interaction between VAPB and p-α-syn, which also disappeared upon inhibition of phosphorylation (Figure 5C).

We then investigated whether this interaction was specific to phosphorylation-modified *α*-syn by examining the binding of unphosphorylated h-α-syn with VAPB. CO-IP analysis using a h-α-syn-specific antibody demonstrated that h-α-syn interacts with VAPB in both TG mouse brain tissues and cellular models (Figures 5D,E). Overexpression of VAPB further confirmed its interaction with h-*α*-syn (Figure 5F). These findings suggest that both p-α-syn and h-α-syn interact with VAPB, and this interaction is independent of phosphorylation at serine 129.

To further investigate the direct interaction between p-*α*-syn and PTPIP51/VAPB, we purified recombinant GST-tagged GST-p-*α*-syn and GST-h-α-syn proteins *in vitro*. GST pull-down assay revealed that GST-p-*α*-syn could bind to both PTPIP51 and VAPB, whereas GST-h-α-syn did not interact with PTPIP51 (Figures 5G,H), consistent with the CO-IP results mentioned earlier.

### 2.6 P-*α*-syn disrupts PTPIP51/VAPB interaction

The PTPIP51-VAPB complex serves as a crucial molecular bridge between the ER and mitochondria, forming a physical tether that facilitates inter-organelle communication (Jiang et al., 2024; Stoica et al., 2014). This connection is essential for maintaining intracellular calcium homeostasis and regulating cellular processes such as lipid metabolism and mitochondrial dynamics (Shiiba et al., 2025). Given the pivotal role of the PTPIP51-VAPB complex, we investigated whether p-*α*-syn binding to PTPIP51 and VAPB could modulate their interaction.

**Figure 6:**
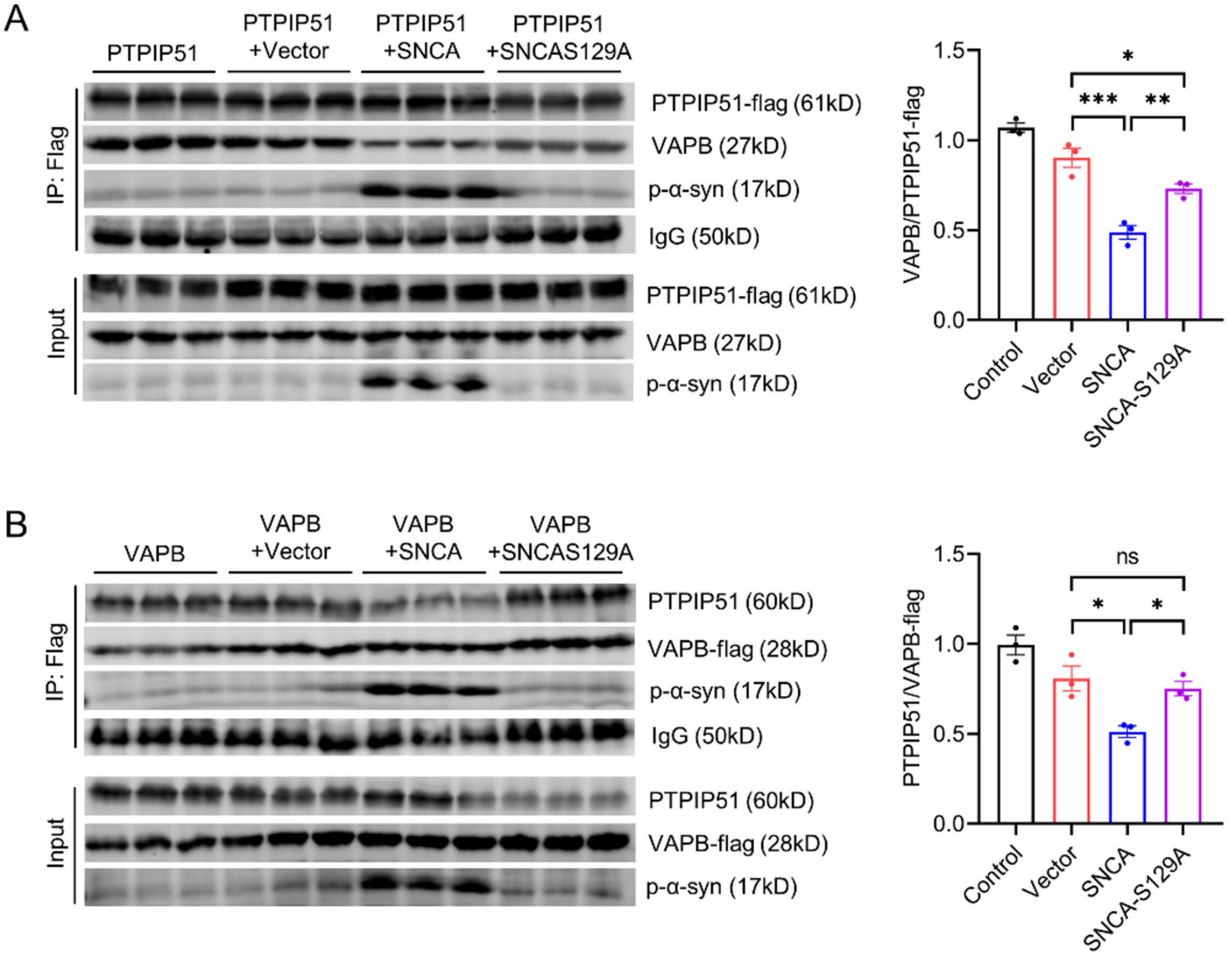
P-α-syn disrupts PTPIP51/VAPB interaction. **(A)** CO-IP analysis of the interaction between PTPIP51 and VAPB in SH-SY5Y cells co-transfected with PTPIP51-Flag and either Vector, SNCA, or SNCA-S129A plasmids for 48 h. IP was performed using a Flag antibody. The graph on the right shows the ratio of VAPB to PTPIP51-Flag in the CO-IP assay, representing the strength of their interaction. **(B)** CO-IP analysis of the interaction between PTPIP51 and VAPB in SH-SY5Y cells co-transfected with VAPB-Flag and either Vector, SNCA, or SNCA-S129A plasmids for 48 h. IP was performed using a Flag antibody. The graph on the right displays the ratio of PTPIP51 to VAPB-Flag in the CO-IP assay, indicating the interaction strength between the two proteins.

To address this, we performed CO-IP assays in SH-SY5Y cells. First, SH-SY5Y cells were transfected with PTPIP51-flag, and CO-IP analysis using an anti-Flag antibody revealed that the presence of p-*α*-syn disrupted the interaction between PTPIP51 and VAPB (Figure 6A). To validate these findings, we conducted a reciprocal experiment by transfecting SH-SY5Y cells with VAPB-flag. Consistent with the initial results, CO-IP analysis using an anti-Flag antibody demonstrated that p-*α*-syn impaired the PTPIP51-VAPB interaction (Figure 6B). These findings indicate that p-*α*-syn disrupts the formation of the PTPIP51-VAPB complex, which may interfere with their cellular functions.

### 2.7 P-*α*-syn disrupts the transfer of calcium from ER stores to mitochondria

**Figure 7:**
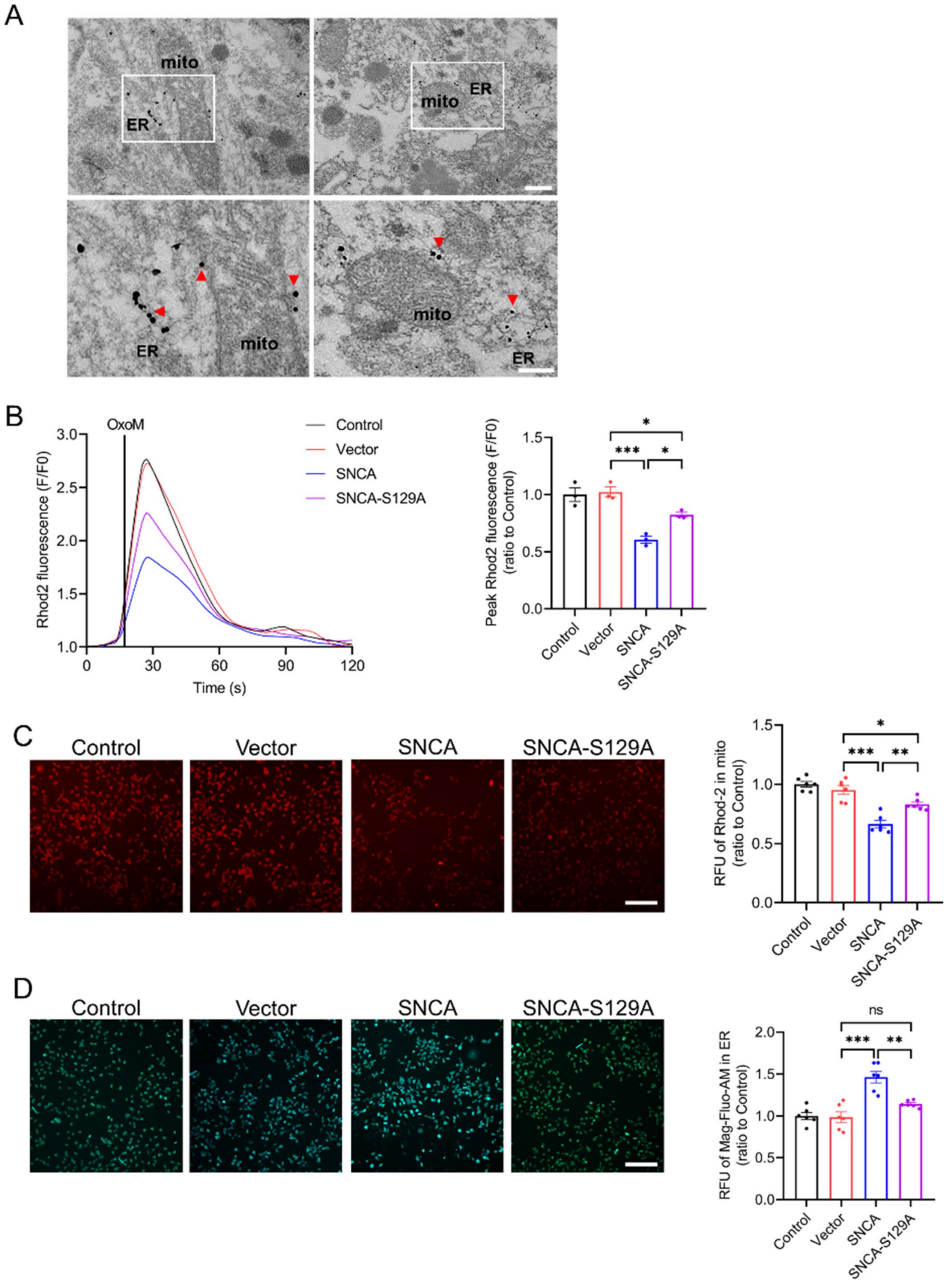
P-α-syn disrupts the transfer of calcium from ER stores to mitochondria. **(A)** Electron microscopy with immunogold labeling showing p-α-syn deposition on the mitochondrial membrane and near the ER in the midbrains of TG mice. Red arrows indicate p-α-syn localization. The white square highlights the area shown at higher magnification. Scale bar: 500 nm (above), 250 nm (below). **(B)** Mitochondrial calcium uptake in SH-SY5Y cells transfected with Vector, SNCA, or SNCA-S129A plasmids for 48 h, following IP3R-mediated release from ER stores. Calcium release was triggered with oxotremorine-M (OxoM), and mitochondrial calcium levels were measured using the Rhod2 calcium probe. Right: Peak values (F/F0) of Rhod2 fluorescence traces, showing mitochondrial calcium uptake (*n* = 3). **(C)** Representative images of mitochondrial calcium levels (red fluorescence) after IP3R-mediated calcium release. Scale bar: 100 μm. Right: Statistical analysis of average red fluorescence intensity (total fluorescence intensity normalized per cell) for mitochondrial calcium levels (*n* = 6). **(D)** ER calcium concentration (green fluorescence) measured in SH-SY5Y cells using the Mag-Fluo-AM probe following IP3R-mediated calcium release. Scale bar: 100 μm. Right: Statistical analysis of average green fluorescence intensity (total fluorescence intensity normalized per cell) for ER calcium levels. Data are presented as mean ± standard error of the mean (SEM). Statistical analysis was performed using one-way ANOVA (*p < 0.05, **p < 0.01, ***p < 0.001).

The interaction of p-α-syn with PTPIP51 and VAPB may impact their function. To explore this, we started by examining the subcellular localization of p-*α*-syn. Immunogold labeling electron microscopy showed that p-*α*-syn aggregates on the mitochondrial membrane and near the ER (Figure 7A). Co-localization analyses using immunofluorescence confirmed that p-*α*-syn co-localizes with PTPIP51 (Supplementary Figure S1A) and VAPB (Supplementary Figure S1B), consistent with prior CO-IP results. Additionally, we found that elevated p-α-syn levels do not affect the expression levels of PTPIP51 or VAPB (Supplementary Figure S2).

Given that PTPIP51 and VAPB form a transmembrane complex facilitating IP3R-mediated calcium transfer between the ER and mitochondria (De Vos et al., 2012), we hypothesized that p-*α*-syn disrupts this process. To test this, SH-SY5Y cells were transfected with Vector, SNCA, or SNCA-S129A plasmids and stimulated with oxotremorine-M (oxoM) to induce IP3R-mediated calcium release (De Vos et al., 2012; Stoica et al., 2014; Stoica et al., 2016). Overexpression of h-*α*-syn reduced mitochondrial calcium uptake and delayed ER–mitochondrial calcium exchange, while inhibiting phosphorylation at Ser129 partially restored these functions (Figure 7B). High-content imaging with the Rhod-2-AM probe further confirmed that h-*α*-syn overexpression reduced mitochondrial calcium levels following IP3R activation, whereas phosphorylation inhibition restored them (Figure 7C). Conversely, ER calcium levels measured by Mag-Fluo-AM increased with h-*α*-syn overexpression but decreased when phosphorylation was inhibited (Figure 7D). These findings suggest that p-*α*-syn disrupts calcium transfer from ER to mitochondria, while inhibition of phosphorylation restores calcium homeostasis.

## 3 Discussion

**Figure 8:**
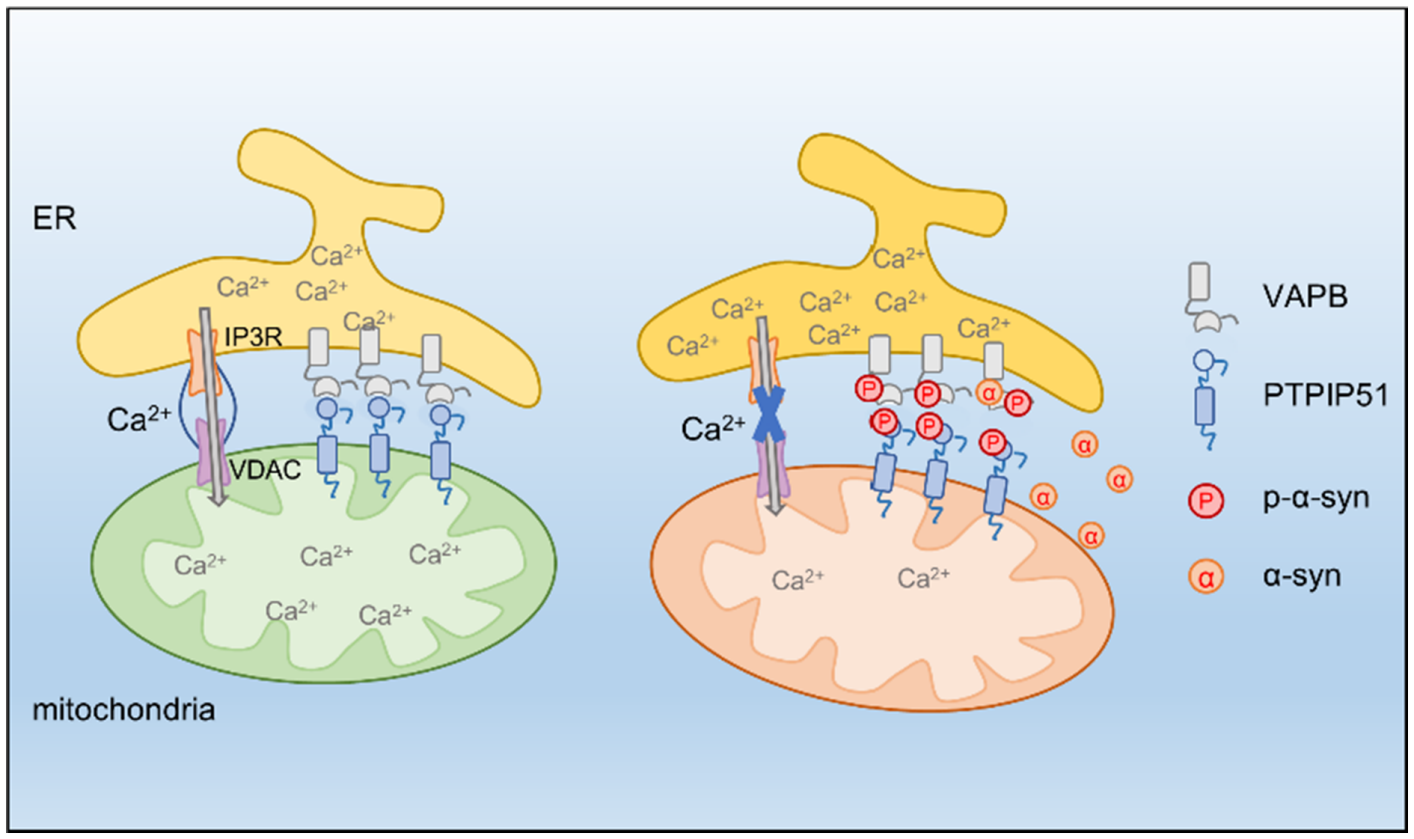
PTPIP51 and VAPB form a tethering complex at the MAM, promoting calcium transfer from the ER to mitochondria. P-α-syn interacts with PTPIP51 and VAPB, destabilizing this complex and disrupting ER-mitochondria calcium signaling, ultimately leading to mitochondrial dysfunction. Inhibiting α-syn phosphorylation restores calcium homeostasis and improves mitochondrial function.

In this study, we identified p-α-syn as a mediator of mitochondrial dysfunction and calcium dysregulation in PD. By interacting with PTPIP51 and VAPB, p-α-syn disrupts ER-to-mitochondria calcium transfer, impairing mitochondrial function. These findings provide new insights into the molecular mechanisms underlying p-*α*-syn toxicity and highlight the potential of targeting Ser129 phosphorylation as a therapeutic approach (Figure 8).

Phosphorylation at Ser129 is the prominent post-translational modification (PTM) of *α*-syn, with abnormal accumulation of p-*α*-syn serving as a hallmark of PD (Hassanzadeh et al., 2024; Anderson et al., 2006). Previous studies have implicated p-*α*-syn in mitochondrial dysfunction, oxidative stress, and inflammation, ultimately contributing to neurodegeneration (Kawahata et al., 2022; Butler et al., 2022; Kam et al., 2022). However, contrasting evidence suggests that *α*-syn phosphorylation may play a protective role by increasing solubility and reducing the formation of toxic oligomers, particularly during early disease stages (Ramalingam et al., 2024). Our findings contribute to this controversy by showing a pathogenic role for p-*α*-syn in mitochondrial dysfunction. Using a phosphorylation-deficient mutant (SNCA-S129A), we showed that inhibiting Ser129 phosphorylation reduces ROS levels and improves mitochondrial function, including complex I activity, ATP production, and mitochondrial respiration. These observations underscore the importance of Ser129 phosphorylation in mediating *α*-syn toxicity.

To explore the molecular basis of p-*α*-syn-induced mitochondrial damage, we employed CO-IP/MS analysis, which identified PTPIP51 and VAPB as key interacting partners of p-*α*-syn. VAPB, a conserved ER transmembrane protein, mediates inter-organelle communication by facilitating ER-mitochondria tethering (Obara et al., 2024; Phillips and Voeltz, 2016). A mutation in VAPB associated with sporadic PD, characterized by a missing valine residue at position 25, has been shown to impair its function and contribute to cellular dysfunction (Kun-Rodrigues et al., 2015). Previous studies have reported that *α*-syn destabilizes the VAPB-PTPIP51 tether by interacting with VAPB, impairing calcium signaling and reducing mitochondrial ATP production (Erustes et al., 2021; Paillusson et al., 2017). However, the specific role of p-α-syn in this process has remained unclear. Our findings address this gap, revealing that while both α-syn and p-α-syn interact with VAPB, only p-α-syn uniquely binds to PTPIP51. This distinctive interaction suggests a separate pathogenic mechanism for p-α-syn. Specifically, the p-α-syn-PTPIP51 interaction may underlie the exacerbation of mitochondrial dysfunction, setting it apart from the role of non-phosphorylated *α*-syn.

Furthermore, p-α-syn weakens the PTPIP51-VAPB complex. Our results indicate that elevated p-α-syn levels impair PTPIP51-VAPB interactions, potentially leading to functional deficits. Beyond disrupting PTPIP51-VAPB binding, p-α-syn may also contribute to calcium dysregulation through additional mechanisms. First, PTPIP51 is associated with the mitogen-activated protein kinase (MAPK) signaling pathway, which plays a key role in cellular stress responses and calcium signaling (Dietel et al., 2018). P-*α*-syn may alter PTPIP51-mediated signaling, thereby indirectly affecting calcium homeostasis. Second, p-α-syn may interfere with PTPIP51-VAPB interactions with other essential proteins, further disrupting calcium transfer. These possibilities warrant further investigation.

We further observed that overexpression of h-*α*-syn, which increases both h-α-syn and p-α-syn levels, disrupts ER-to-mitochondria calcium transfer, leading to reduced mitochondrial calcium uptake and delayed calcium exchange. Inhibiting Ser129 phosphorylation restored calcium transfer and improved mitochondrial calcium levels, emphasizing the critical role of p-*α*-syn in this process. However, the incomplete recovery suggests that non-phosphorylated α-syn also contributes to calcium transfer deficits, likely through cooperative effects with p-α-syn in impairing the PTPIP51-VAPB complex at MAMs. This highlights the dual role of phosphorylated and non-phosphorylated α-syn in disrupting calcium signaling and mitochondrial function.

Insufficient calcium transfer between the ER and mitochondria not only compromises mitochondrial energy metabolism and ATP production but also disrupts key mitochondrial dynamics, such as fission, fusion, and autophagy (Casas-Martinez et al., 2024; Daverkausen-Fischer and Pröls, 2022). Additionally, impaired calcium transfer contributes to oxidative stress, loss of mitochondrial membrane potential, and activation of apoptotic pathways, collectively exacerbating neuronal damage (Yong et al., 2019). These downstream effects deserve further validation in future studies.

While our study provides evidence for p-*α*-syn-mediated mitochondrial dysfunction, several limitations should be addressed. First, our findings are primarily based on mouse and cellular models, and further validation in human PD samples is needed. Second, while we identified PTPIP51 and VAPB as critical interacting partners, other proteins and pathways likely contribute to p-*α*-syn toxicity (Grassi et al., 2018; Jiao et al., 2024) and warrant further exploration.

In conclusion, our study highlights the importance of Ser129 phosphorylation in α-syn toxicity and suggests that p-α-syn may play a collaborative role in driving mitochondrial dysfunction in PD. While these findings contribute to a deeper understanding of PD pathophysiology, further research will be essential to fully elucidate the mechanisms involved. We hope that this work provides a valuable starting point for exploring therapeutic strategies targeting p-*α*-syn to mitigate neurodegeneration in PD.

## 4 Materials and methods

### 4.1 Animals

C57BL/6 WT and h-α-syn-overexpressing TG mice (strain 017682) were purchased from The Jackson Laboratory (Bar Harbor, ME, USA). All mice were housed at the Laboratory Animal Center of Capital Medical University (Beijing, China) under controlled conditions, maintaining a temperature range of 22–25°C with a 12-h light/dark cycle. All experimental protocols were conducted in accordance with the Animal Care and Use Guidelines of the National Institutes of Health (Bethesda, MD, USA) and were approved by the Institutional Animal Care and Use Committee (Approval Number: AEEI-2020-017).

### 4.2 Western blot analysis

Western blot analysis was performed using primary antibodies, including anti-p-α-syn (D1R1R; Cell Signaling Technology, Danvers, MA, USA), anti-h-α-syn (ab138501; Abcam, Cambridge, UK), anti-VAPB (1477-1-AP; Proteintech, Chicago, IL, USA), anti-PTPIP51 (20641-1-AP; Proteintech), and anti-*β*-actin (66009-1-Ig; Proteintech). Protein concentrations from cells and brain tissue lysates were measured using a BCA Protein Assay Kit (Thermo Scientific). Proteins were separated on 12% SDS-PAGE gels and transferred onto PVDF membranes (Millipore). Membranes were blocked with 5% skim milk for 1 h at room temperature, incubated with primary antibodies overnight at 4°C, and washed three times with TBST. Secondary antibody incubation was performed for 1 h at room temperature, and signals were visualized using an Odyssey imaging system (LI-COR Biosciences, Lincoln, NE, USA).

### 4.3 Co-immunoprecipitation (CO-IP)

Brain tissue (2 mg) or cell lysates (1 mg) were incubated overnight at 4°C with anti-h-α-syn (Abcam), anti-p-α-syn (Cell Signaling Technology), or anti-flag (C1305; Applygen Technologies Inc.) antibodies. Magnetic beads (Med Chem Express, San Rafael, CA, USA) were pre-washed with IP buffer (150 mM NaCl, 10 mM Tris–HCl [pH 7.5], 2 mM EDTA, 0.5% Triton X-100). The antibody-protein mixture was added to the beads and incubated for 6 h at 4°C. Following three washes with IP buffer, antigen–antibody complexes were eluted with loading buffer, heated at 95°C for 10 min, and analyzed by western blotting.

### 4.4 Immunofluorescence

The following primary antibodies were used for immunofluorescence: anti-p-α-syn (Wako, Osaka, Japan), anti-h-α-syn (ab138501; Abcam), anti-TH (ab76442; Abcam), anti-VAPB (1477-1-AP; Proteintech), and anti-PTPIP51 (20641-1-AP; Proteintech). Brain sections were initially incubated with PBST (0.3% Triton X-100 in 0.01 M PBS) for 10 min to permeabilize the tissue. Afterward, the sections were blocked with 5% goat serum (5,424; Cell Signaling Technology) for 1 h to reduce non-specific binding. The slices were then incubated with the primary antibodies at 4°C for 24 h. Following three washes with PBST, the sections were treated with secondary antibodies at room temperature for 1 h, and counterstained with DAPI (D9542; Sigma-Aldrich) for 15 min. Imaging was performed using a Leica SP8 confocal microscope (Solms, Germany).

### 4.5 Pre-embedding immunoelectron microscopy

Mice were deeply anesthetized and perfused with a 0.9% NaCl solution to remove residual blood, followed by perfusion of the circulatory system with a fixative mixture containing 4% paraformaldehyde (PFA) and 0.075% glutaraldehyde. The brain was then removed, fixed for 8 h in a solution of 4% PFA and 0.2% glutaraldehyde, and sliced into 50 μm sections. These brain slices were first incubated for 1 h in PBST, followed by blocking with 5% goat serum for 1 h. The sections were then incubated with the C140S antibody at 4°C for 24 h (Liu et al., 2022). After three washes in PBST containing 1% BSA, the sections were incubated for 1 h with 1.4 nm gold-conjugated anti-rabbit and anti-mouse IgG secondary antibodies. Following three additional washes, the sections were post-fixed in 2% glutaraldehyde. Silver enhancement (Nanoprobes, 2013) was applied for 5 min, and the sections were washed three times with deionized water. Finally, immunogold-labeled ultrathin sections were examined using a JEM-2100 transmission electron microscope (JEOL, Tokyo, Japan).

### 4.6 Cell culture and transfection

SH-SY5Y neuroblastoma cells were cultured in Dulbecco’s Modified Eagle Medium (DMEM; Gibco, Carlsbad, CA, USA) supplemented with 10% fetal bovine serum (FBS; Gibco) to maintain cell growth and proliferation. The cells were incubated at 37°C in a humidified atmosphere containing 5% CO₂ to ensure optimal conditions for cell survival. For transfection, plasmid DNA was introduced into the SH-SY5Y cells using polyethylenimine (PEI) transfection reagent (Polysciences, Inc., Warrington, PA, USA), specifically the linear form with a molecular weight of 25,000 (catalog number 23966). The transfection process was carried out following the manufacturer’s protocol. Briefly, the plasmids were mixed with PEI in serum-free DMEM to form DNA-PEI complexes, which were then added to the cells. After a specified incubation period, the cells were typically harvested for downstream applications, such as protein expression analysis or further experimental procedures.

### 4.7 Primary neuronal culture

All procedures involving animals were approved by the Institutional Animal Care and Use Committee of Capital Medical University and were conducted in compliance with the NIH Guide for the Care and Use of Laboratory Animals. The experiments were designed and executed following strict ethical guidelines to ensure the humane treatment of animals. Primary neuronal cultures were derived from the brains of mouse embryos, typically between embryonic days E15 and E18. To prepare the neurons, pregnant mice were first anesthetized with chloral hydrate. After proper anesthesia was achieved, the embryos were harvested, and the brains were carefully dissected in a sterile environment. The brain tissues were then dissociated using enzymatic treatment, followed by mechanical trituration to obtain a single-cell suspension of neurons. The dissociated neurons were plated onto glass cover slips that were pre-coated with 0.1 mg/mL poly-L-lysine (P1524; Sigma-Aldrich) to promote cell adhesion. The culture medium used for neuron growth was Neurobasal medium (21103–049; Gibco), which is specifically designed for supporting the survival and differentiation of primary neurons. The medium was supplemented with 0.5 mM L-glutamine and B27 supplement (17504–044; Gibco) to provide essential nutrients and promote neuronal health and growth. The neurons were cultured at 37°C in a 5% CO₂ atmosphere, with the medium replaced every 2–3 days to maintain optimal growth conditions.

### 4.8 Measurement of ER and mitochondrial calcium

The measurement of ER calcium was conducted using Mag-Fluo4 acetoxymethyl ester (Mag-Fluo4-AM; Molecular Probes) as part of a cell ER calcium detection assay kit (GMS10267.1 v.A; GENMED, Shanghai, China). Similarly, mitochondrial calcium levels were assessed using Mag-Fluo4-AM (Molecular Probes) within a cell mitochondrial calcium detection assay kit (GMS10153.1 v.A; GEN-MED). Fluorescence changes were monitored using Cellomics high content screening (HCS; Thermo Scientific) at excitation/emission wavelengths of 488/530 nm (green) or 549/595 nm (red).

### 4.9 Measurement of mitochondrial complex I activity

Mitochondrial complex I activity was measured according to the manufacturer’s protocol using the Mitochondrial Complex I Activity Assay Kit (AAMT001-1KIT; Merck-Millipore, Darmstadt, Germany). Protein content in cell and tissue samples was quantified using the Bicinchoninic Acid (BCA) Protein Assay Kit (23,225; Pierce Biotechnology, Rockford, IL, USA), following three freeze–thaw cycles to lyse the cells and tissues. The activity of complex I was assessed by measuring the ratio of (NAD)H to NAD^+^, with an increase in absorbance at 450 nm recorded after a 30-min incubation. For further analysis, cell and tissue lysates were incubated for 3 h at room temperature with a specific mitochondrial complex I antibody. The enzymatic activity was quantified by calculating the change in absorbance at 450 nm per minute, which reflects the rate of complex I activity.

### 4.10 ATP level measurement

ATP levels in cultured cells were assessed using an ATP assay kit (G9241, Promega, Madison, WI, USA). Cells (1 × 10^4^) were plated in opaque 96-well black plates and treated as per the experimental requirements. The plate was allowed to reach room temperature for about 30 min before measurement. Next, 100 μL of ATP detection buffer was added to each well, followed by a 2-min mixing to lyse the cells. The plate was incubated at room temperature for 10 min to stabilize the luminescent signal. Luminescence was then measured using a microplate reader.

### 4.11 JC-1 staining

Mitochondrial membrane potential (MMP) was assessed using the JC-1 probe (T4069; Sigma-Aldrich). Under low MMP conditions, JC-1 exists as monomers that fluoresce green, while at high MMP, it aggregates and emits red fluorescence. Cells were cultured in 24-well plates, washed three times with PBS, and incubated with JC-1 at 37°C for 30 min. Fluorescence changes were monitored using Cellomics High Content Screening (HCS; Thermo Scientific). Fluorescence intensity was measured at two emission wavelengths: 530 nm (green) and 595 nm (red), and the MMP was evaluated by calculating the ratio of green to red fluorescence intensity.

### 4.12 Cell viability assay

Cell viability was evaluated using the CCK-8 assay, which relies on the WST-8 and PMS components. A total of 1 × 10^4^ cells were seeded into the wells of a 96-well plate and transfected with the plasmid for 24 h. The CCK-8 reagent was diluted 1:10 with culture medium, and 100 μL of the diluted solution was added to each well. The plate was incubated at 37°C, and absorbance was measured at 450 nm using a microplate reader (PerkinElmer, Inc., Waltham, MA, USA) every h. The absorbance value at the second h was used for analysis in this study.

### 4.13 LDH assay

Cytotoxicity was measured using the LDH assay. Briefly, 1 × 10^4^ cells were plated into the wells of a 96-well microplate and transfected with plasmids for 24 h. Afterward, 100 μL of the cell supernatant from each well were transferred to a new 96-well plate. LDH detection solution (100 μL) was then added to each well, thoroughly mixed, and incubated in the dark at room temperature (25°C) for 30 min. Following incubation, 50 μL of stop solution was added to each well, and the mixture was gently agitated for 10 min. Absorbance was measured at 490 nm using a microplate reader (PerkinElmer, Inc., Waltham, MA, USA).

### 4.14 Mitochondrial stress measurement using the XFe-24 seahorse assay

The oxygen consumption rate (OCR) of SH-SY5Y cells was assessed using a Seahorse XFe analyzer (Seahorse Bioscience, USA). Cells were seeded into XFe 24-well plates, and the assay medium was prepared by supplementing Seahorse XFe Base Medium (minimal DMEM, Seahorse Bioscience, USA) with 2 mM L-glutamine, 1 mM pyruvate, and 10 mM glucose (Sigma, USA), adjusted to pH 7.4. For calibration, probes (Seahorse Bioscience, USA) were hydrated in 1 mL of calibrant solution (Seahorse Bioscience, USA) and incubated overnight at 37°C. Before starting the assay, the culture medium was replaced with the prepared assay medium, and plates were incubated in a non-CO2 incubator at 37°C for 45 min. During the Mito Stress Test, SH-SY5Y cells were treated sequentially with 1.5 μM oligomycin, 1 μM FCCP, and a combination of 0.5 μM antimycin A and rotenone.

### 4.15 Statistical analysis

All statistical analyses were conducted using Prism 8.0.1 software (GraphPad, La Jolla, CA, USA). Each experiment was independently repeated at least three times. Results are presented as the mean ± standard error of the mean (SEM), and statistical significance was defined as *p* < 0.05.

## Data Availability

The original contributions presented in the study are included in the article/Supplementary material, further inquiries can be directed to the corresponding author.
