# Supplementary material for: Serine-129 phosphorylated α-synuclein drives mitochondrial dysfunction and calcium dysregulation in Parkinson’s disease model
Source: Front Aging Neurosci. 2025 Mar 31;17:1538166. doi: 10.3389/fnagi.2025.1538166 (PMC11994663; doi:10.3389/fnagi.2025.1538166)
Supplement: Supplementary file 1 [file Table_1.docx]

**Figure S1**


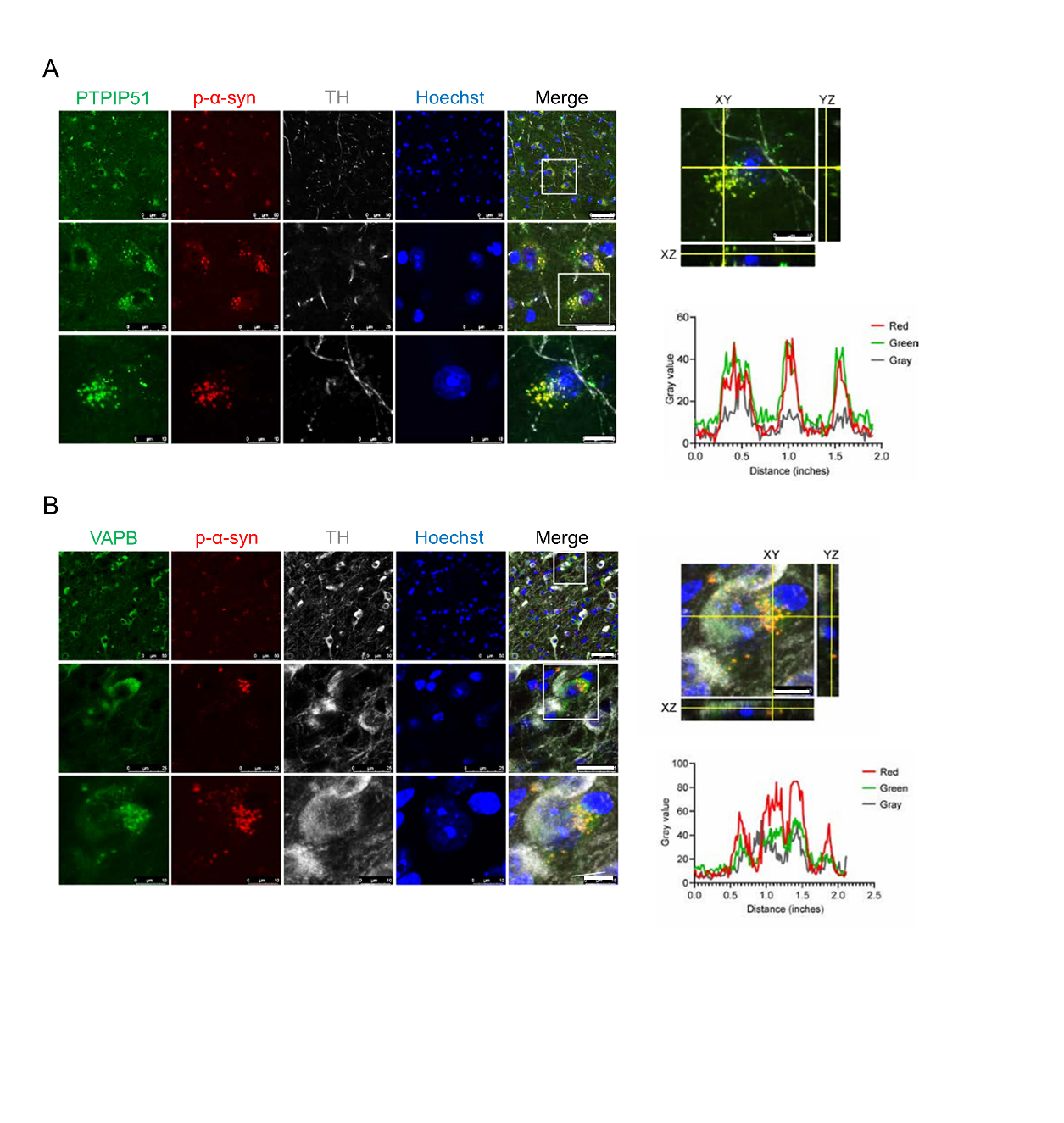


Figure S1. Co-localization of p-α-syn with PTPIP51 and VAPB in the midbrains of TG mice. (A, B) Immunofluorescence analysis showing the expression and co-localization of PTPIP51 (A) and VAPB (B) with p-α-syn in the midbrains of transgenic (TG) mice. PTPIP51 or VAPB (green), p-α-syn (red), dopaminergic neuron marker tyrosine hydroxylase (TH, gray), and hoechst (blue) are labeled. Above: Low-magnification images of the midbrain regions. Below: High-magnification overlay images highlighting co-localization. White squares in low-magnification images indicate the areas shown in high-magnification views. The orthogonal view (top right) further confirms the co-localization of p-α-syn with PTPIP51 or VAPB. Co-localization analysis was performed using ImageJ software (bottom right). Scale bars: 50 μm (a), 25 μm (b), and 10 μm (c, orthogonal view).

**Figure S2**


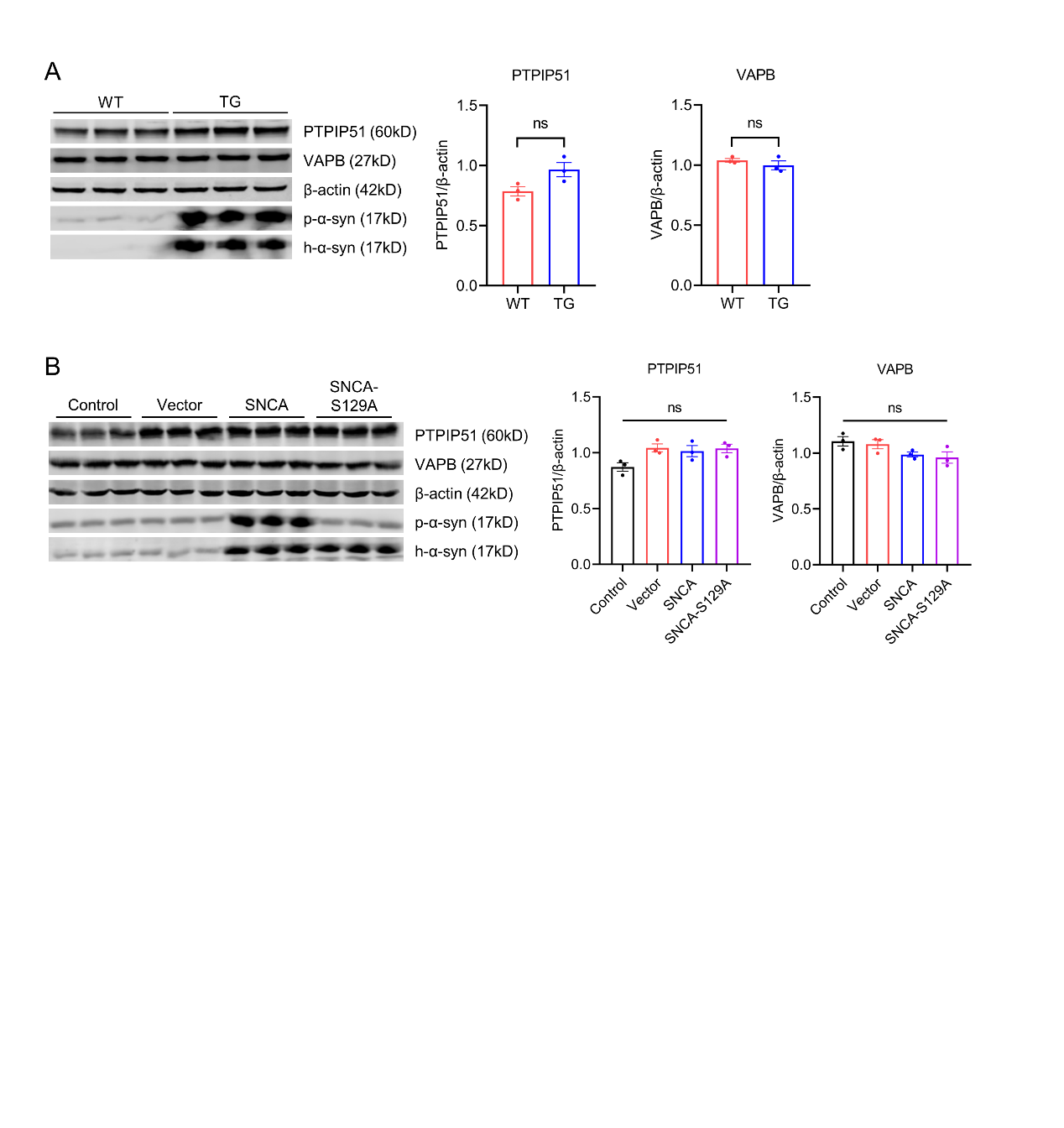


Figure S2. Expression of PTPIP51 and VAPB in mice and SH-SY5Y cells. (A) Western blot analysis of PTPIP51, VAPB, h-α-syn, and p-α-syn expression in the midbrain of WT and TG mice. β-actin was used as a loading control. The graph on the right shows the quantitative analysis of PTPIP51 and VAPB expression, with β-actin used as a loading control (n = 3). (B) SH-SY5Y cells were transfected with Vector, SNCA, or SNCA-S129A plasmids for 48 hours. Representative immunoblot of PTPIP51, VAPB, h-α-syn, and p-α-syn from lysates of SH-SY5Y cells. The graph on the right shows the quantitative analysis of PTPIP51 and VAPB expression, with β-actin as a loading control (n = 3).
